# Inter­esting hurdles in the solution of pharmaceutical crystal structures using powder diffraction data

**DOI:** 10.1107/S2056989026009382

**Published:** 2026-09-11

**Authors:** James A. Kaduk

**Affiliations:** a Illinois Institute of Technology, 3101 S. Dearborn St., Chicago IL 60616, USA; b North Central College, 131 S Loomis St Naperville IL 60540 USA; Harvard University, USA

**Keywords:** pharmaceutical crystal structures, powder diffraction

## Abstract

Although solving and refining a crystal structure using synchrotron powder diffraction data can be routine, many problems present features which make the process more difficult. These include difficulty in indexing, incorrect or approximate symmetry, and chemically unreasonable models (poor conformations, incorrect compounds). Examples of each of these ‘inter­esting’ features are provided, as well as the methods used for recognizing and overcoming them. Raw data and GSAS-II project files are provided for all of the examples.

## Abstract 2

Although solving and refining a crystal structure using synchrotron powder diffraction data can be routine, many problems present features which make the process more difficult. These include difficulty in indexing, incorrect or approximate symmetry, and chemically-unreasonable models (poor conformations, incorrect compounds). Examples of each of these ‘inter­esting’ features are provided, as well as the methods used for recognizing and overcoming them.

## Introduction

Of the approximately 200 pharmaceutical crystal structures I have solved in recent years using synchrotron radiation, about half were straightforward. That is, routine application of available tools yielded a crystal structure that may be regarded as ‘correct’. The other half present a variety of ‘inter­esting’ challenges, which make the structure solution more difficult. In this paper I present a few examples (so that the problems can be more easily recognized) and present some ways to overcome these problems. The problems fall into common classes, so I have grouped them under indexing difficulties, ambiguous determination of the symmetry, and chemical surprises.

These examples come from a project (Kaduk *et al.*, 2014 ▸) to determine the crystal structures of large-volume commercial pharmaceuticals, and include high-quality powder diffraction data for them in the Powder Diffraction File (Kabekkodu *et al.*, 2024 ▸).

## Indexing

Synchrotron powder patterns do not (generally) suffer from the systematic errors in peak positions (mainly displacement and transparency), which can occur in laboratory Bragg–Brentano measurements. Therefore, when a synchrotron pattern cannot be indexed, it’s a sign that something inter­esting is going on.

### Defactinib

Defactinib, C_20_H_21_F_3_N_8_O_3_S, is approved as a treatment for ovarian cancer. The pattern was measured at beam line 11-BM (Lee *et al.*, 2008 ▸; Wang *et al.*, 2008 ▸, Antao *et al.*, 2008 ▸) of the Advanced Photon Source at Argonne National Laboratory.

The pattern was difficult to index. Both laboratory and a previous synchrotron pattern, measured at the Canadian Light Source (Leontowich *et al.*, 2021 ▸), yielded a primitive monoclinic unit cell with *a* = 16.356, *b* = 5.556, *c* = 14.422 Å, *β* = 111.58°, *V* = 1218.72 Å^3^, and *Z* = 4. The space group seemed to be *P*2_1_, but an acceptable structure solution and fit to the pattern were not obtained.

**Figure 1:**
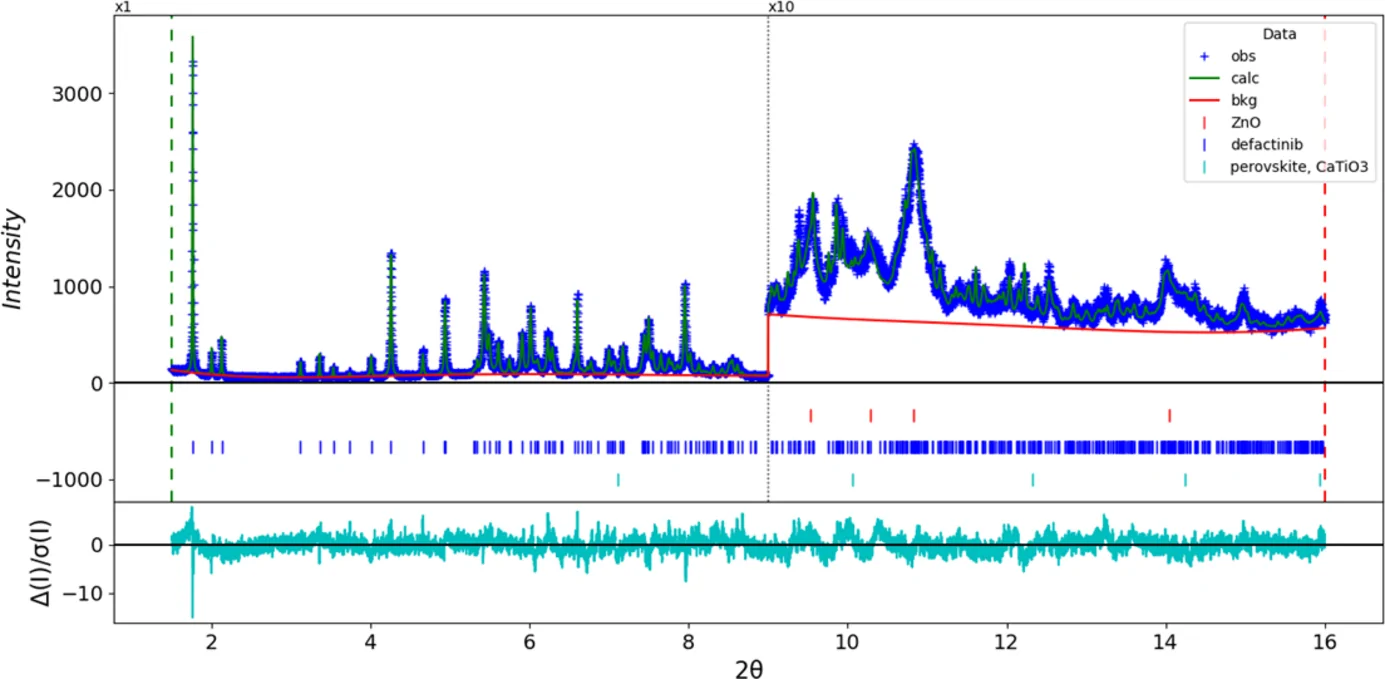
The Rietveld plot for defactinib. The blue crosses represent the observed data points, and the green line is the calculated pattern. The cyan curve is the normalized error plot, and the red line is the background curve. The blue tick marks indicate the defactinib peak positions, the red tick marks indicate the ZnO impurity peak positions, and the cyan tick marks indicate the perovskite impurity peak positions. The vertical scale has been multiplied by a factor of 10 × for 2θ > 9.0°.

The 11-BM pattern was indexed on a primitive monoclinic unit cell with *a* = 17.3551, *b* = 5.1655, *c* = 25.4517 Å, *β* = 97.750°, *V* = 2262.60 Å^3^, and *Z* = 4 with *DICVOL14* (Louër & Boultif, 2014 ▸), using 30 peaks, a larger than usual number. This is an example of a ‘short axis’ problem, and the high resolution of 11-BM certainly helped indexing. The first significant peak with a contribution from the *b* axis is the 15th peak (110) at 5.42°; all of the low-angle peaks have indices *h0l* (Figs. 1 ▸ and 2 ▸; Kaduk *et al.*, 2026*a* ▸).

### Bromfenac sodium sesquihydrate Form I

Bromfenac sodium, Na(C_15_H_11_BrNO_3_), (marketed as Prolensa, Bromday, and Yellox) is used for the management of ocular pain and treatment of postoperative inflammation in patients who have undergone cataract extraction. Powder diffraction data for bromfenac sodium Form I (sesquihydrate) and Form III (anhydrous) are reported in United States Patent 8,299,295 B2 (Matharu *et al.*, 2012 ▸; Johnson Matthey Public Limited Company).

The pattern, measured at 11-BM, was difficult to index. Several indexing programs suggested a triclinic cell [M/F(20) = 134.9/1129.5] with *a* = 4.6337, *b* = 18.3323, *c* = 20.3382 Å, *α* = 98.61, *β* = 93.68, *γ* = 92.31°, and *V* = 1702.11 Å^3^. Given the bromfenac formula of C_15_H_12_BrNO_3_ and the usual assumption of 18 Å^3^/non-H atom, we expect the unit-cell volume to be an integral multiple of 360 Å^3^. A value of *Z* = 4 implies a cell volume of 1440 Å^3^, so this cell volume is unexpectedly large.

**Figure 3:**
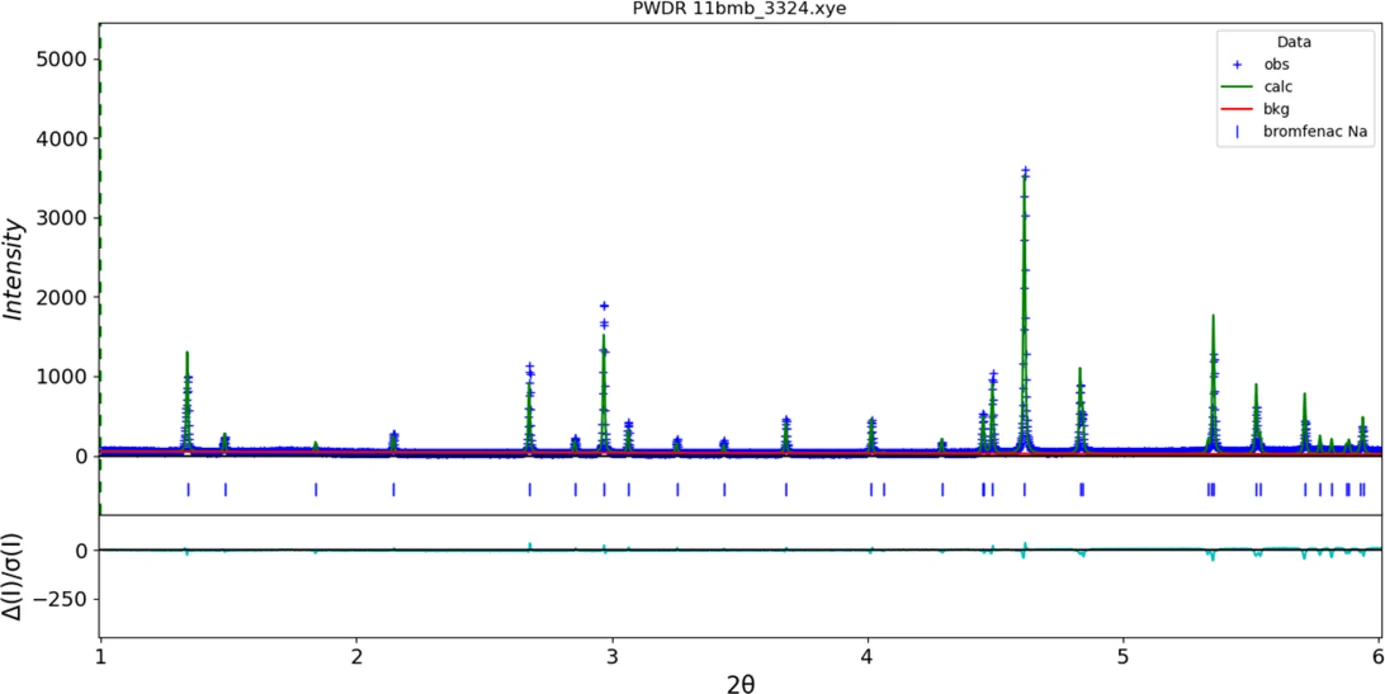
The low-angle portion of a Le Bail fit to the powder pattern of bromfenac sodium sesquihydrate using the first unit cell. The blue crosses represent the observed data points, and the green line is the calculated pattern. The cyan curve is the normalized error plot, and the red line is the background curve. The blue tick marks indicate the peak positions.

**Figure 4:**
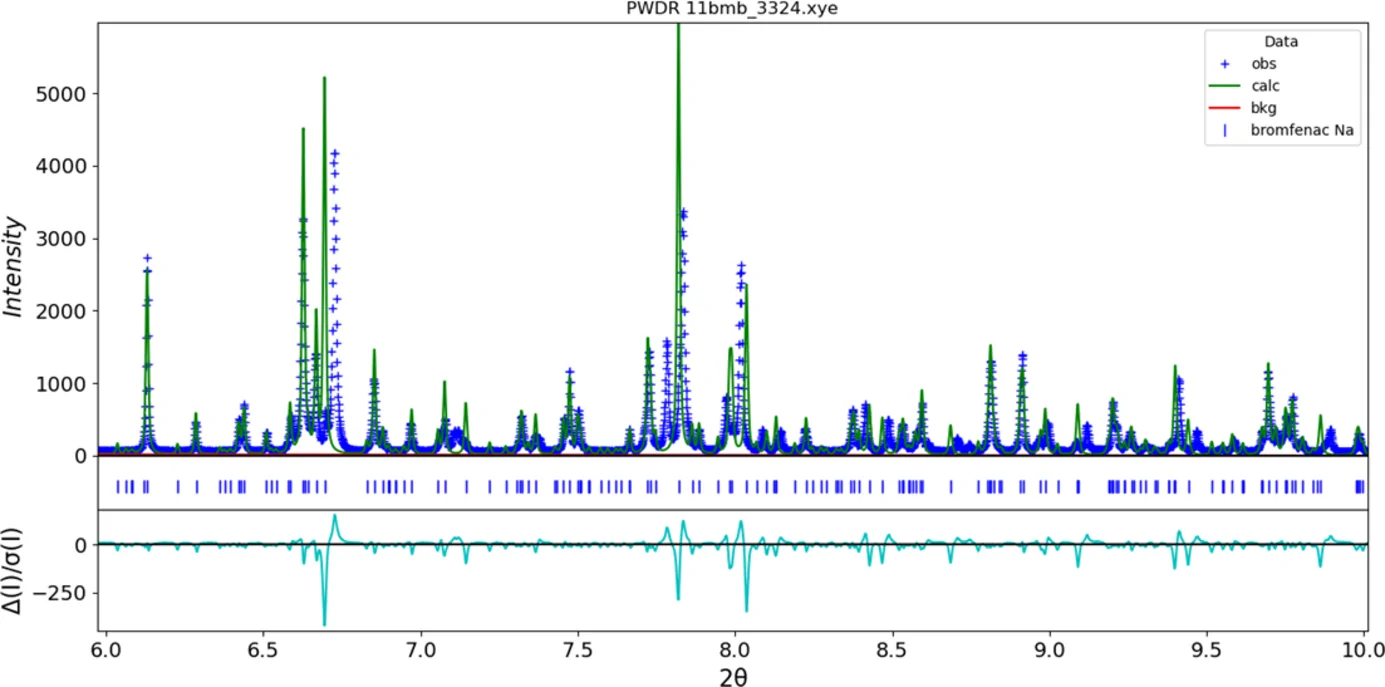
A higher-angle portion of the Le Bail fit to the pattern of bromfenac sodium sesquihydrate, showing that the first unit cell did not account for the higher-angle peak peaks.

**Figure 5:**
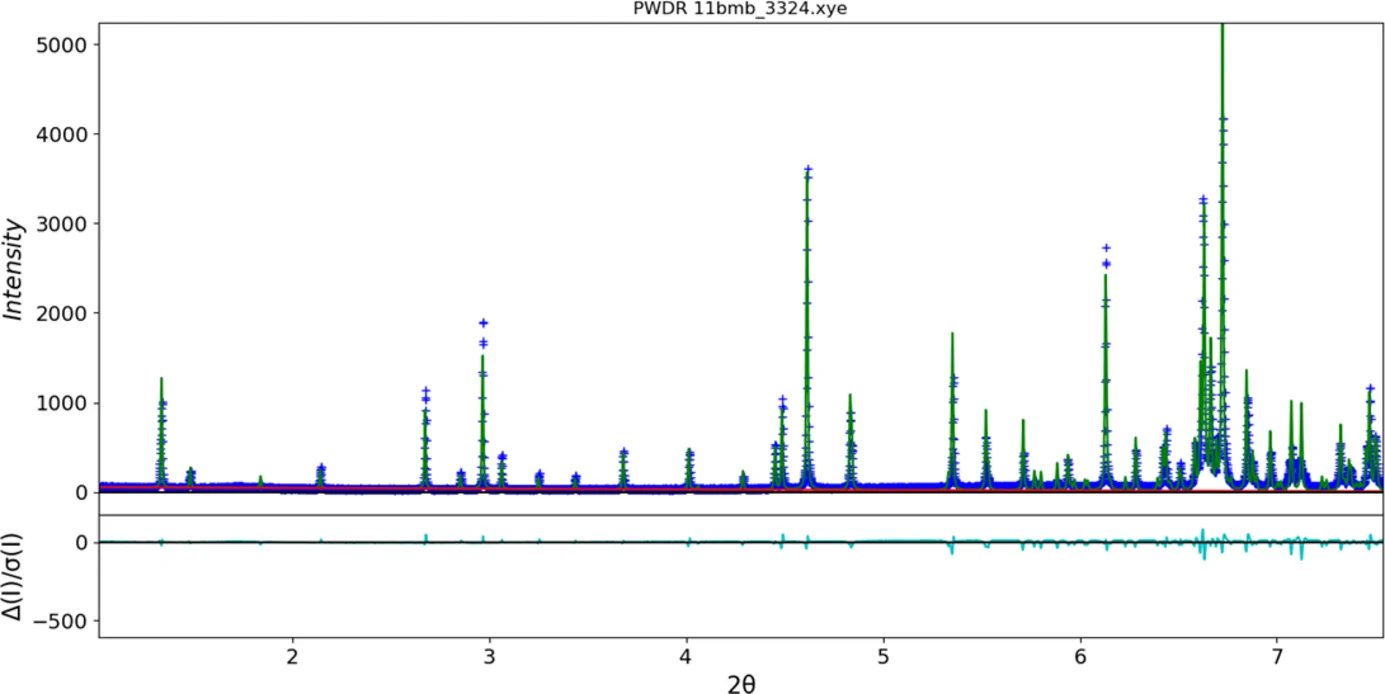
The low-angle portion of a Le Bail fit to the powder pattern of bromfenac sodium sesquihydrate using the second unit cell.

**Figure 6:**
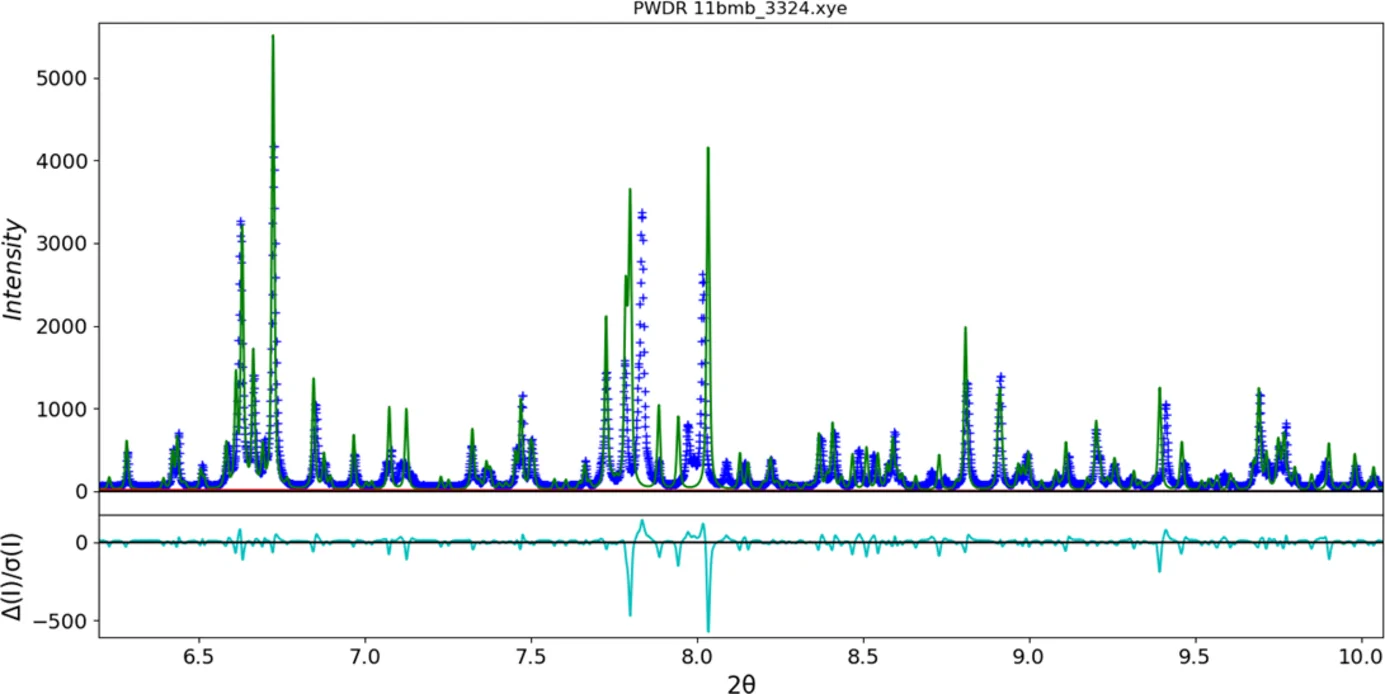
A higher-angle portion of the Le Bail fit to the pattern of bromfenac sodium sesquihydrate, showing that the second unit cell did not account for the higher-angle peak peaks.

A Le Bail fit modeled the low-angle peaks well (Fig. 3 ▸), but the fit became poor at ‘high angles’ > 6° 2θ (Fig. 4 ▸). Expanding the number of peaks from 20 to 38 and using *DICVOL14* (Louër & Boultif, 2014 ▸) led to a triclinic cell (M/F = 386/3723) with *a* = 4.6756, *b* = 18.3116, *c* = 20.3818 Å, *α* = 81.233, *β* = 84.818, *γ* = 89.826°, and *V* = 1717.51 Å^3^. The Le Bail fit was good out to 7.5° 2θ (Fig. 5 ▸), but then became poor at higher angles (Fig. 6 ▸). Expanding the number of peaks to 62 (to 8.3°) led *DICVOL14* [both natively and through the *PreDict* inter­face (Blanton *et al.*, 2019 ▸)] to suggest a triclinic cell (M/F = 386/3723) with *a* = 4.1772, *b* = 18.3119, *c* = 20.3123, *α* = 98.767, *β* = 92.070, *γ* = 90.408°, and *V* = 1534.47 Å^3^. This cell had a more reasonable volume, explained the positions of all the peaks, and we believe is the correct cell. A reduced cell search in the Cambridge Structural Database (version 2026.1.0; Groom *et al.*, 2016 ▸) yielded no hits.

This is thus another example of a ‘short axis’ problem. The first peak with any contribution from the *a* axis is the 32nd peak (100) at 6.43°, but this peak is overlapped. The first distinct peak with an *h* contribution is the 35th peak (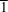
01) at 6.58°. It is thus not surprising that a larger-than-usual number of peaks had to be used for the indexing.

The structure was solved using direct methods as implemented in *EXPO2014* (Altomare *et al.*, 2013 ▸), applying the COVMAP extensions to examine all trials. One of the solutions yielded two almost complete bromfenac mol­ecules. Only the O atoms of the carboxyl­ate groups, one carbonyl C atom, and one of the phenyl rings had to be edited manually. Seven additional atoms were present in the solution.

Initial refinement (with heavily restrained bromfenac mol­ecules) of the positions and occupancies of these seven atoms yielded two atoms with low (*frac* < 0.2) occupancies and which moved too close to the bromfenac anions. These two atoms were deleted. Analysis of the inter­atomic distances and occupancies of the remaining five atoms suggested that two of them were Na, and the other three were O (water mol­ecules). The powder pattern is similar enough to that reported for bromfenac sodium sesquihydrate Form I (Matharu *et al.*, 2012 ▸) to suggest that they represent the same material, assuming that the patent pattern exhibits significant preferred orientation. The compound thus appears to be bromfenac sodium sesquihydrate (Salazar *et al.*, 2026*a* ▸).

## Symmetry

### Xanomeline hydrogen tartrate

Xanomeline hydrogen tartrate, (C_14_H_24_N_3_OS)(HC_4_H_4_O_6_), is being developed to treat schizophrenia The powder pattern, measured at 298 (1) K at the Wiggler Low Energy Beamline (Leontowich *et al.*, 2021 ▸) of the Brockhouse X-ray Diffraction and Scattering Sector of the Canadian Light Source, was indexed using *JADE Pro* (MDI, 2025 ▸) on a primitive monoclinic unit cell with *a* = 7.49890, *b* = 37.49914, *c* = 7.46736 Å, *β* = 89.90°, *V* = 2099.84 Å^3^, and *Z* = 4. The suggested space group was *P*2_1_/*n*, but since both xanomeline and tartrate are chiral, this is impossible. The *β* angle close to 90° suggested that the true symmetry might be ortho­rhom­bic. This possibility was tested by a Le Bail fit using *EXPO2014* (Altomare *et al.*, 2013 ▸). The suggested space group was *P*2_1_2_1_2_1_ (consistent with the chiral constituents), which was confirmed by successful solution and refinement of the structure (Kaduk *et al.*, 2026*b* ▸).

## Chemical reasonableness

### Daprodustat Form 1

Daprodustat, C_19_H_27_N_3_O_6_, (marketed as Duvroq, among others) is used to treat anemia due to chronic kidney disease. Crystalline Forms 3 and 4 of daprodustat are claimed in Inter­national Patent Application WO 2020/102302 A1 (Šamec *et al.*, 2020 ▸; Teva Pharmaceuticals Inter­national GmbH). Powder patterns are reported for these two forms, as well as for Form 1 and a mixture of Form 1 and Form 2.

**Figure 7:**
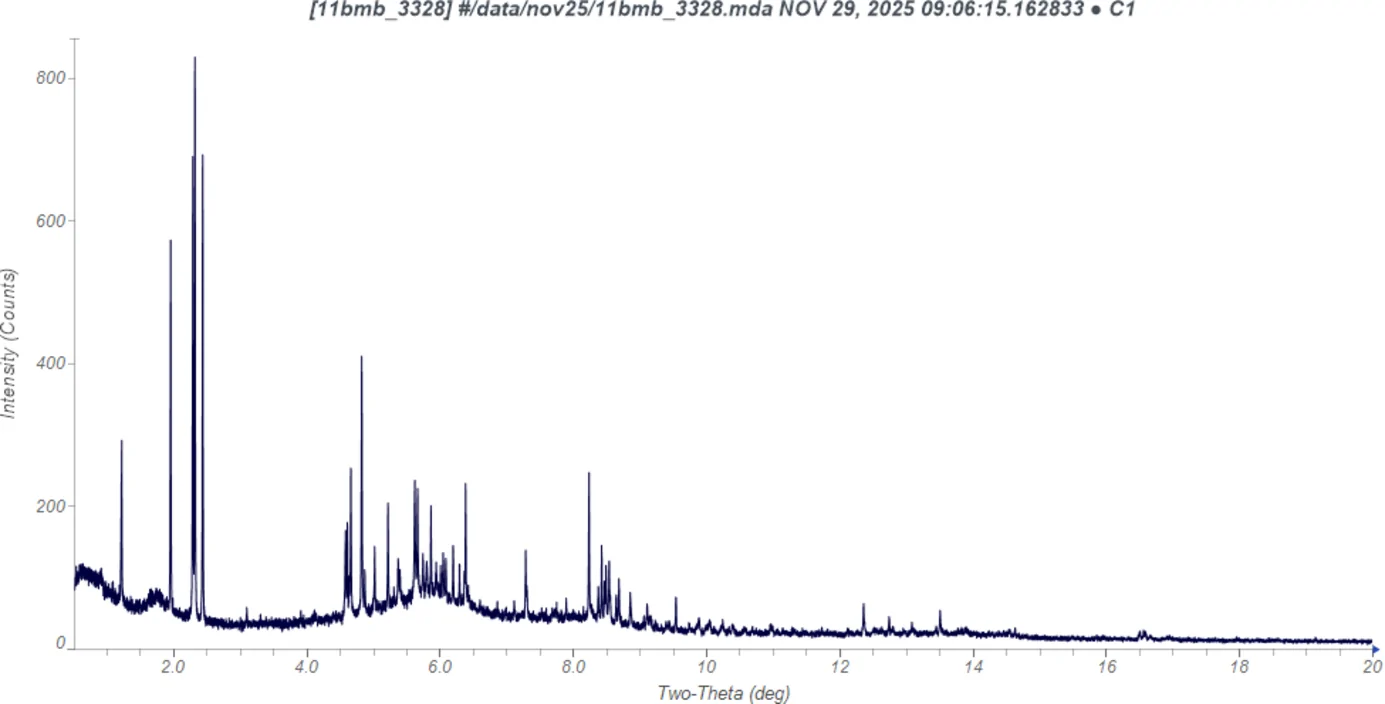
The synchrotron powder pattern of daprodustat, measured at beamline 11-BM at APS using a wavelength of 0.4687342 Å.

This pattern, measured at 11-BM, is much less intense than usual (825 counts in the strongest peak, compared to 10-20,000 for other pharmaceuticals measured on 11-BM; Fig. 7 ▸). The scattering of the Kapton capillary is much more prominent than usual. The peaks are quite sharp (FWHM ∼0.010°, compared to the instrumental FWHM ∼0.0065°), so the sample seems highly crystalline. In this batch of samples, two measurements were of empty capillaries, and one was from an empty sample position. I believe that this daprodustat specimen was the residual sample clinging to the walls of the capillary after the majority fell out.

The pattern was indexed on a primitive triclinic unit cell with *a* = 6.53817, *b* = 13.76411, *c* = 22.22234 Å, *α* = 88.99, *β* = 83.92, *γ* = 88.84°, *V* = 1987.92 Å^3^ using *JADE Pro* (MDI, 2025 ▸). The volume corresponds to *Z* = 4, so there are two independent mol­ecules in the structure. The space group was assumed to be *P*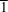, which was confirmed by successful solution and refinement of the structure. The structure was solved using Monte Carlo simulated annealing techniques as implemented in *EXPO2014* (Altomare *et al.*, 2013 ▸). One of the ten runs yielded an *R*_wp_ (14.2%) much less than the others (≥ 20%), so the success rate was low.

**Figure 8:**
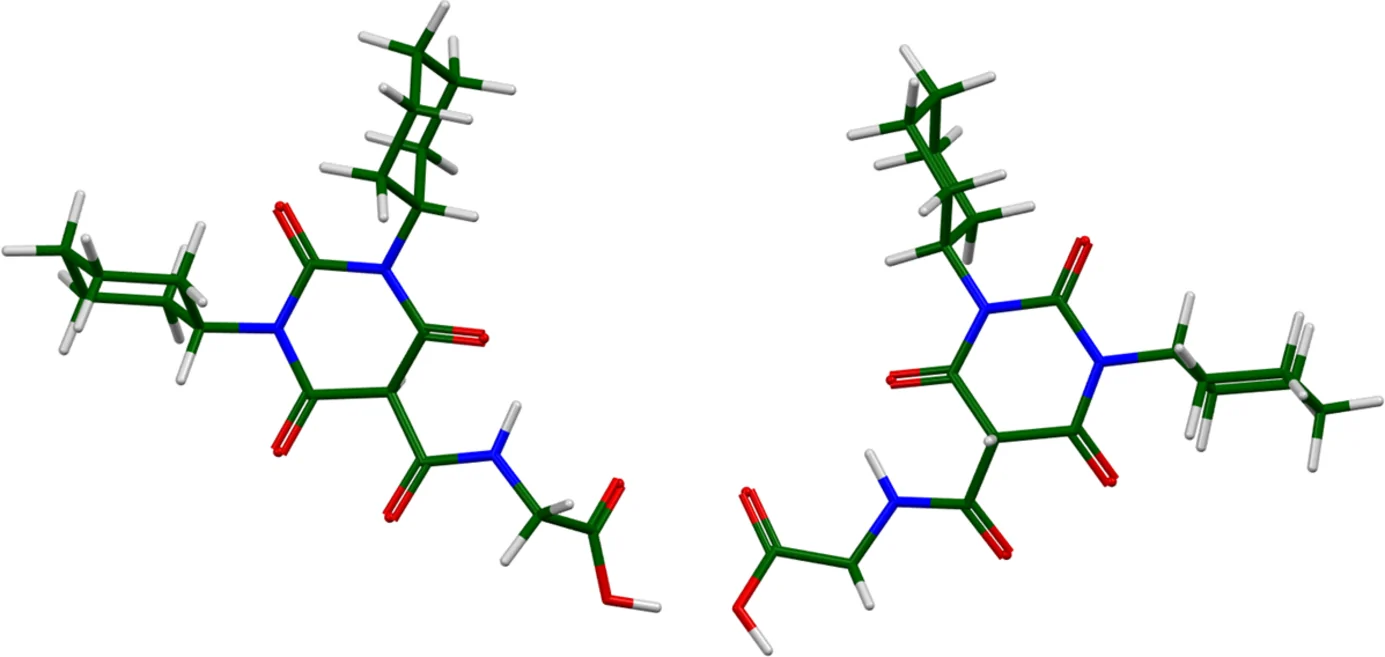
The initial structure model of daprodustat, illustrating the unreasonable orientations of the carb­oxy­lic acid groups.

**Figure 9:**
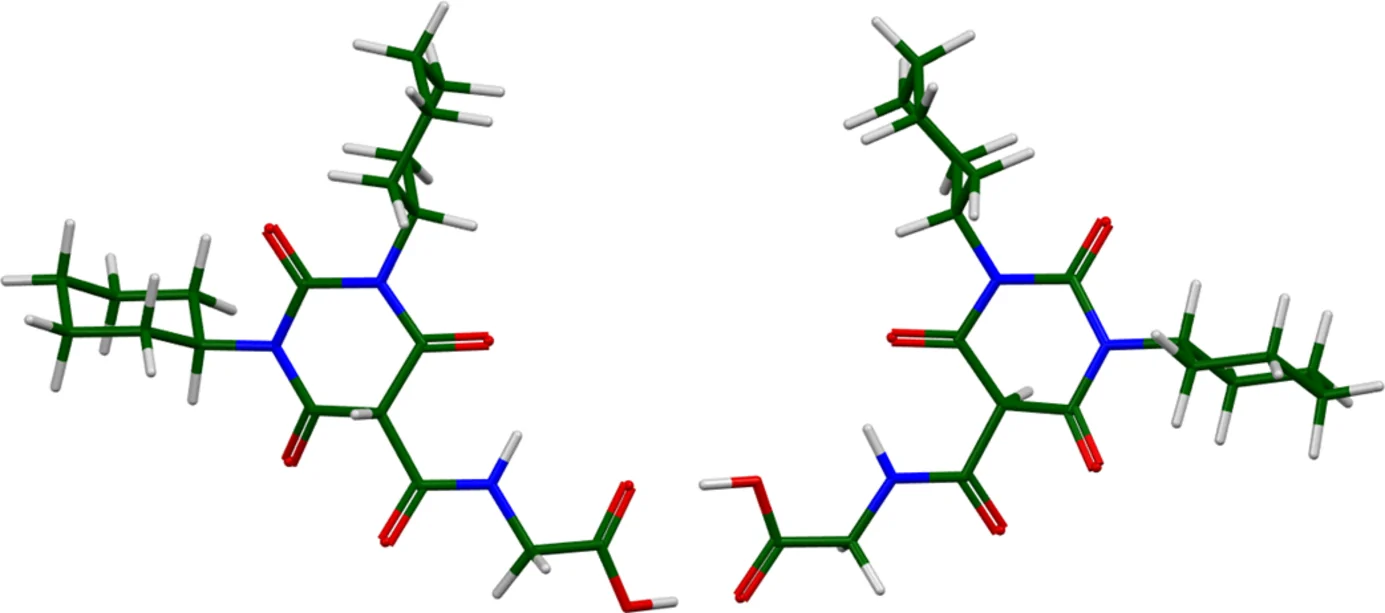
The lower-energy (and correct) structure model for daprodustat.

In the initial structure model, the carb­oxy­lic acid groups of the two mol­ecules were oriented to form the usual dimer, with graph set (Etter, 1990 ▸; Bernstein *et al.*, 1995 ▸; Motherwell *et al.*, 2000 ▸) 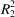
(8), but the carbonyl groups were on the same side of the ring (Fig. 8 ▸) – a chemically unreasonable result. Accordingly, the carb­oxy­lic acid group in each mol­ecule was separately rotated by 180° using *Materials Studio* (Dassault Systèmes, 2025 ▸). The resulting two structures were optimized using *VASP* (Kresse & Furthmüller, 1996 ▸). The structure with the carboxyl group of mol­ecule 1 rotated was 32.4 kcal mol^−1^/cell lower in energy (Fig. 9 ▸), so was adopted for the final refinement (Salazar *et al.*, 2026*b* ▸).

### Resmetirom heminona­hydrate Form CSI

Resmetirom, C_17_H_12_Cl_2_N_6_O_4_, (marketed under the trade name Rezdiffra) is a thyroid hormone receptor-β (THR-β) agonist. It is approved by the FDA (U.S. Food and Drug Administration) as the first treatment of liver fibrosis for adults with non-cirrhotic non-alcoholic steatohepatitis (NASH). A new crystalline form of resmetirom, Form CSI, was claimed in Inter­national Patent Application WO 2021/063367 A1 [Chen & Chang, 2021 ▸; Crystal Pharmaceutical (Suzhou) Co. Ltd.], and powder data (no crystal structure) are provided. Thermogravimetric data provided in Example 2 of WO 2021/063367 A1 indicate that Form CSI is a dihydrate. The powder pattern of this study corresponded to Form CSI.

The powder pattern, measured at 298 (1) K at the Wiggler Low Energy Beamline (Leontowich *et al.*, 2021 ▸) of the Brockhouse X-ray Diffraction and Scattering Sector of the Canadian Light Source, was difficult to index, suggesting that the sample was not a single phase. After many failures using multiple programs, the successful strategy was to use *N-TREOR* as incorporated into *EXPO2014* (Altomare *et al.*, 2013 ▸), permitting up to three unindexed lines. The pattern was indexed on a primitive triclinic unit cell with *a* = 11.29881*, b* = 15.12763, *c* = 16.49587 Å, *α* = 67.695, *β* = 74.517, *γ* = 69.701°, *V* = 2416.7 Å^3^, and *Z* = 4. The space group was assumed to be *P*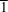, which was confirmed by the successful solution and refinement of the structure.

**Figure 10:**
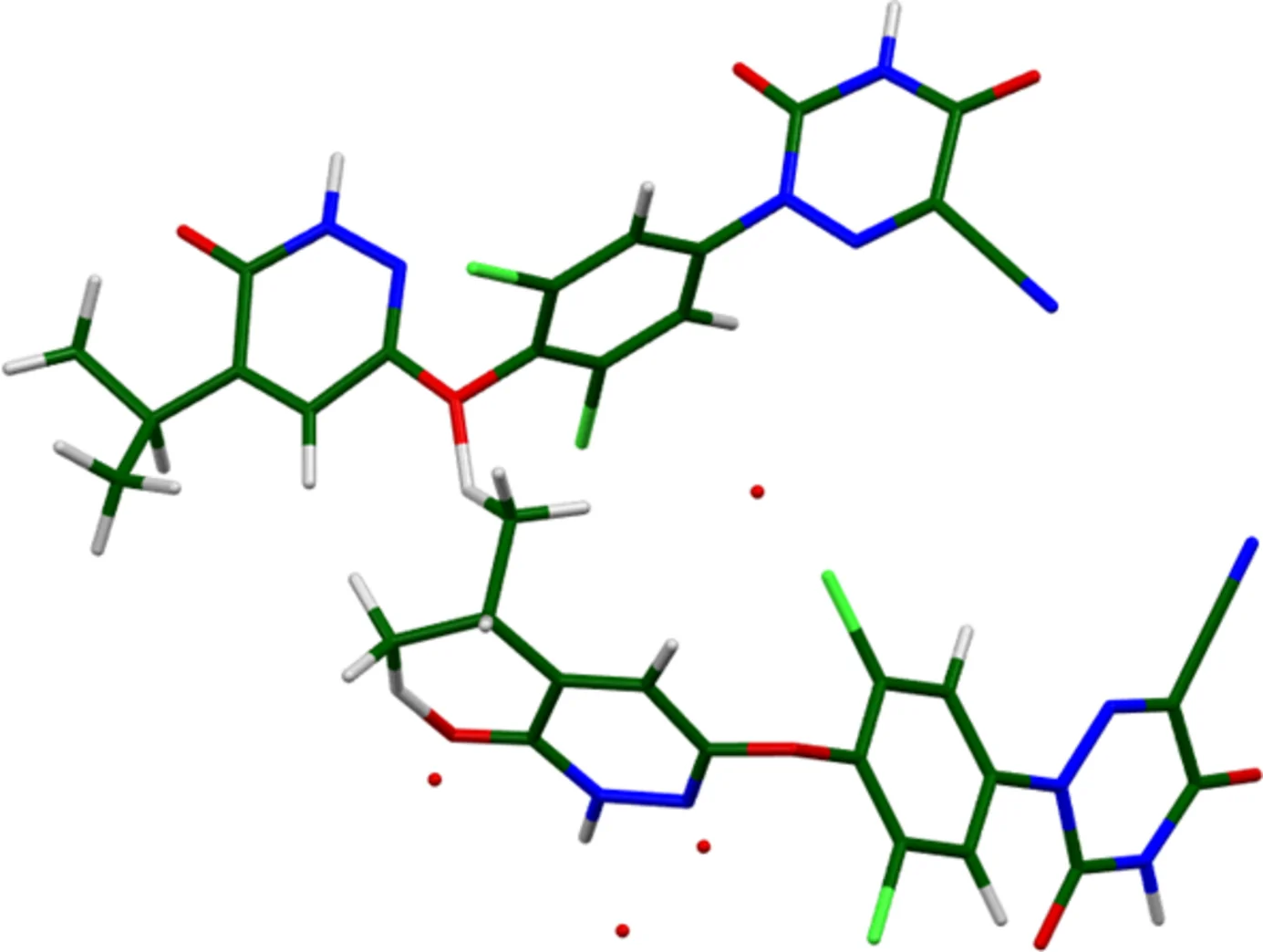
The ‘best’ structure model for resmetirom trihydrate from the Monte Carlo simulated annealing, showing the unreasonable conformation of one of the mol­ecules and close contacts.

**Figure 11:**
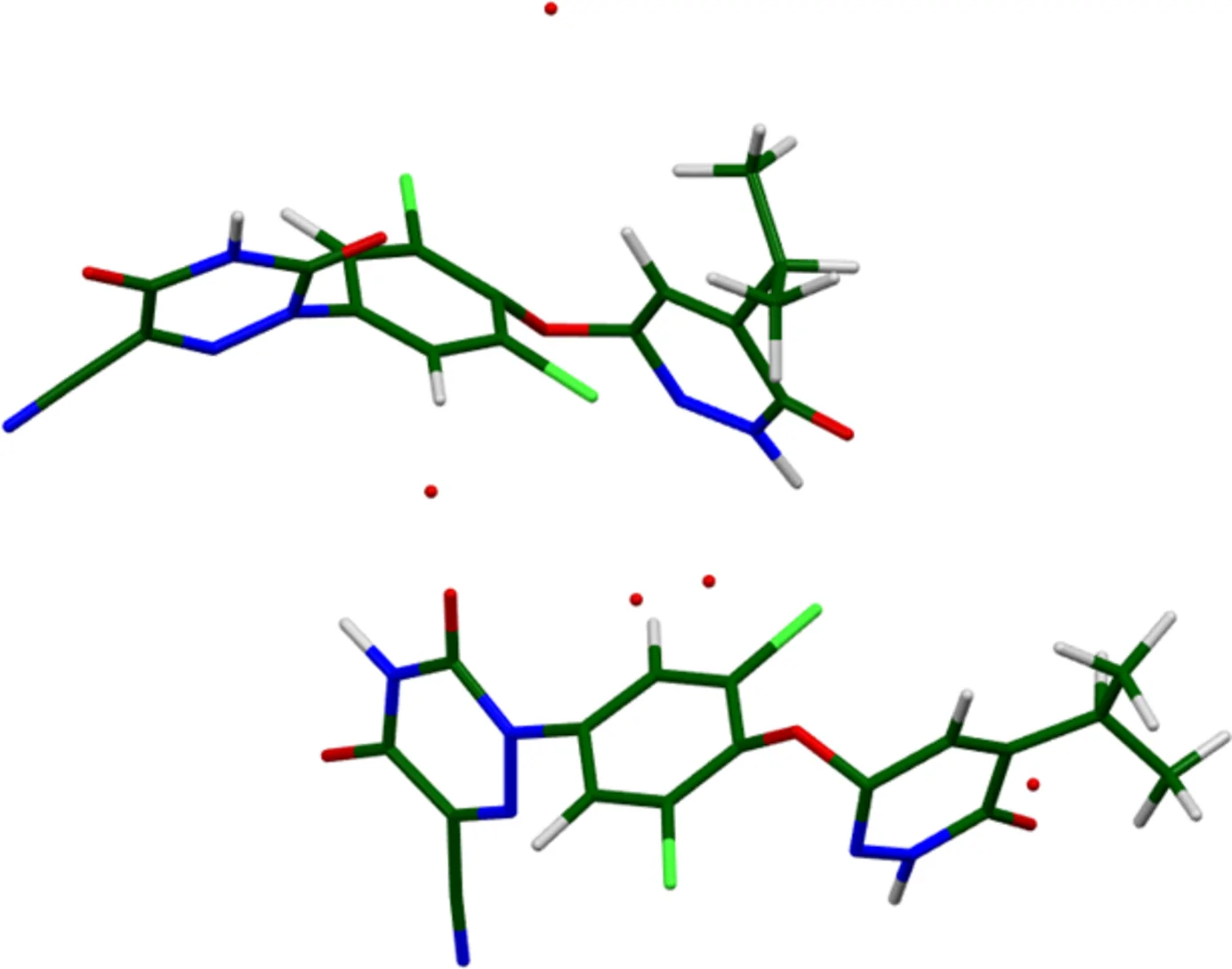
The second-best structure model for resmetirom trihydrate from the Monte Carlo simulated annealing, with reasonable conformations of both mol­ecules.

The crystal structure was solved by Monte Carlo simulated annealing techniques as implemented in *EXPO2014* (Altomare *et al.*, 2013 ▸), including a bump penalty on the non-H atoms. Using two resmetirom mol­ecules and four O atoms (water mol­ecules) yielded solutions that contained voids, so the process was repeated with 6 O atoms. In the best solution (*R*_wp_ = 15.01%) one of the mol­ecules had an unreasonable conformation and there were close contacts (Fig. 10 ▸), but the second-best solution (*R*_wp_ = 15.73%) contained two reasonable mol­ecules (Fig. 11 ▸). Refinement of the O atoms led to one of the O atoms with an occupancy of about 0.25 and too close to the mol­ecules, suggesting that the compound was a hemi­penta­hydrate.

The structure model still contained voids, so the sample was analyzed by NMR, which estimated the presence of 4.7 water mol­ecules per resmetirom molecule. In addition, the triplet signal at 0.86 ppm combined with a multiplet at 1.24 ppm indicated the presence of a *n*-C_11_–C_12_ aliphatic hydro­carbon. The sample contained ∼0.11 *n*-C_11–12_ per resmetirom mol­ecule. The powder pattern indicated that the sample contained a significant amount of an amorphous material; we assume that this phase contains the hydro­carbon. Using the normal rule of thumb of 18 Å^3^/non-H atom, the unit-cell volume of 2427 Å^3^ corresponds to 135 non-H atoms/cell. The resmetirom mol­ecule (C_17_H_12_Cl_2_N_6_O_4_) contains 29 non-H atoms, so *Z* = 4 implies 116 atoms/cell. Thus, the cell volume indicates an additional 19 atoms/cell, or resmetirom(H_2_O)_4.75_.

**Figure 12:**
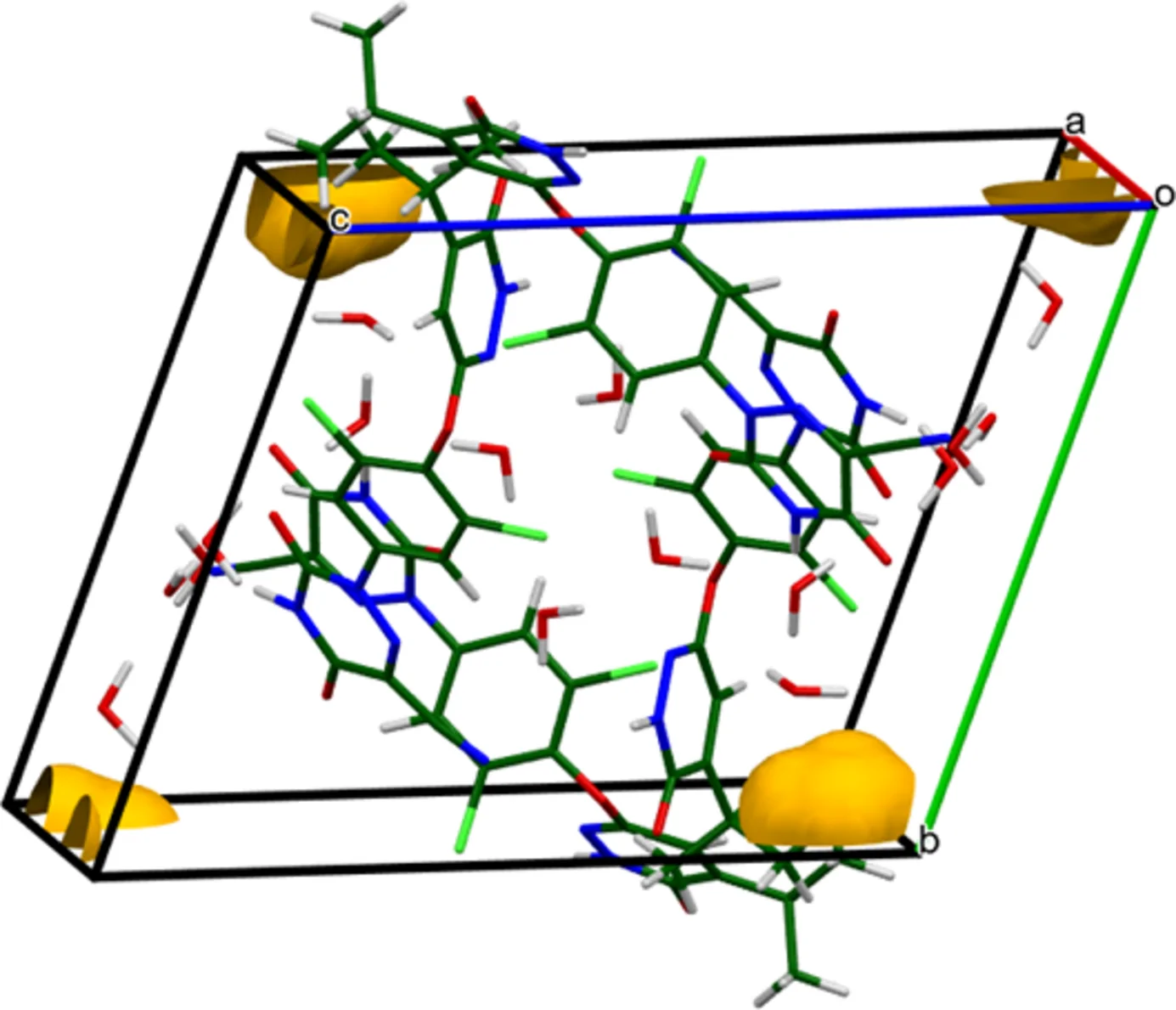
The structure of resmetirom trihydrate, after removing three unreasonable water mol­ecules from the structure solution of resmetirom heminonohydrate.

**Figure 13:**
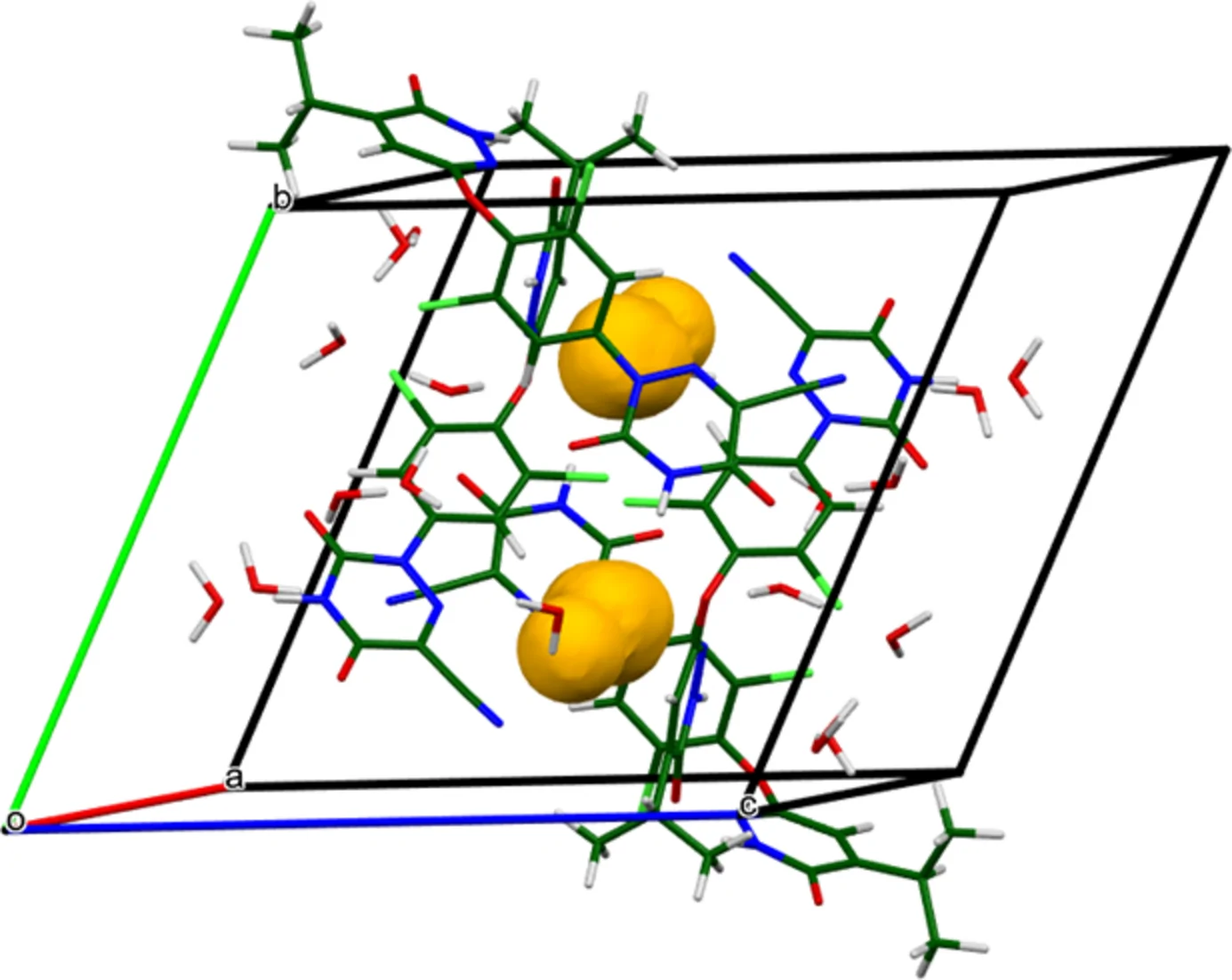
The void after the *VASP* optimization of the structure of resmetirom tetra­hydrate.

To include the water mol­ecules in the structure solution, the structure solution was thus repeated using two resmetirom mol­ecules and nine O atoms (water mol­ecules) as fragments. One of the O atoms was too close to the other atoms, so it was deleted from the model. After initial refinement, two additional O moved too close, and were deleted. There were, however, two voids (Fig. 12 ▸), so O atoms were placed at the centers of the voids and included in the refinement. DFT optimization (after recalculation of the H positions) using *VASP* indicated another void (Fig. 13 ▸), and a ninth O atom was added to the refinement. 18 water mol­ecules/cell resulted in no additional voids, so the compound is apparently a heminona­hydrate (4.5 water/resmetirom) (Kaduk *et al.*, 2025*a* ▸).

### Acoltremon

Acoltremon, C_18_H_27_NO_2_, (sold under the brand name Tryptyr, and also known as AR-15512) is used to treat dry-eye syndrome. The pattern, measured at 11-BM, was indexed on a primitive ortho­rhom­bic unit cell with *a* = 9.32059, *b* = 11.39187, *c* = 16.25977 Å, *V* = 1727.5 Å^3^, and *Z* = 4 using *N-TREOR* as incorporated into *EXPO2014* (Altomare *et al.*, 2013 ▸). The suggested space group was *P*2_1_2_1_2_1_, which was confirmed by successful solution and refinement of the structure. A reduced cell search in the Cambridge Structural Database (version 2026.1.0; Groom *et al.*, 2016 ▸), combined with the chemistry C, H, N, and O only, yielded 22 hits, but no structures for acoltremon or its derivatives.

**Figure 14:**
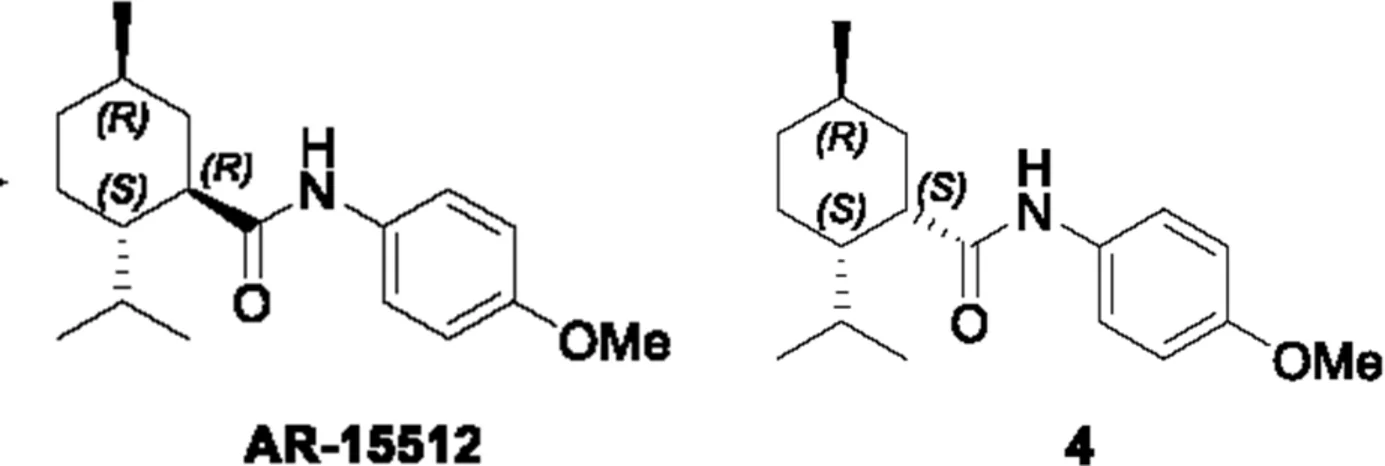
The mol­ecular structure of acoltremon (AR-15512, left) compared to the diastereomer structure determined by Rodriguez-Arévalo *et al.* (2021 ▸). Image from Rodriguez-Arévalo *et al.* (2021 ▸).

A crystal structure of acoltremon at 100 K has been reported (Rodríguez-Arévalo *et al.*, 2021 ▸), but it is of the (1*S*,2*S*,5*R*) diastereomer, and not the (1*R*,2*S*,5*R*) of the active pharmaceutical (Fig. 14 ▸). The *a*, *b* and *c* lattice parameters at 298 K were 2.0% larger, 9.7% larger, and 7.0% smaller, respectively, than those at 100 K. Refinement was begun using the fractional coordinates of Rodriguez-Arévalo *et al.* (2021 ▸), but was unsatisfactory. Then we realized that they were for a different diastereomer. The refinement changed the chiralities to result in the enanti­omer of the correct diastereomer. The structure was solved using Monte Carlo simulated annealing techniques as implemented in *EXPO2014* (Altomare *et al.*, 2013 ▸), using the correct diastereomer as the fragment (Salazar *et al.*, 2026*c* ▸).

### Atrasentan hydro­chloride Form 1

Atrasentan, C_29_H_38_N_2_O_6_, (marketed as VANRAFIA) is used to reduce proteinuria. It is also sold as the hydro­chloride salt. Powder data for anhydrous atrasentan, a quarterhydrate, and a hemihydrate are reported in US Patent US 8,962,675 B1 (Gong & Zhang, 2015 ▸; AbbVie Inc.). Crystalline Form 1 of atrasentan hydro­chloride is claimed in US Patent Application US 2006/0189675 A1 (King, 2006 ▸; Abbott Laboratories), and powder data and a unit cell are reported.

The powder pattern was indexed using *JADE Pro* (MDI, 2025 ▸) on a high-quality (FOM = 4) primitive ortho­rhom­bic unit cell with *a* = 8.00309, *b* = 17.64533, *c* = 21.27799 Å, *V* = 3004.82 Å^3^, and *Z* = 4. The suggested space group was *P*2_1_2_1_2_1_, which was confirmed by successful solution and refinement of the structure. A reduced cell search in the Cambridge Structural Database (version 2026.1.0; Goom *et al.*, 2016 ▸), combined with the chemistry C, H, N, and O only, yielded seven hits, but no structures for atrasentan or its derivatives. Both our powder pattern and unit cell match those of atrasentan hydro­chloride Form 1, so we conclude that our ‘atrasentan’ sample is really atrasentan hydro­chloride.

The structure was solved using Monte Carlo simulated annealing techniques as implemented in *EXPO2014* (Altomare *et al.*, 2013 ▸), using an atrosentan mol­ecule and a Cl atom as fragments, with a bump penalty and 001 preferred orientation. The three best solutions had short inter- and/or intra­molecular contacts, but the fourth best was more chemically reasonable. A hydrogen atom was added to N7 using *Materials Studio* (Dassault Systèmes, 2025 ▸), and Rietveld refinement was begun. The initial residual *R*_wp_ was ∼14%. The hydrogen-atom positions were recalculated using *Materials Studio*, and the structure was optimized using *VASP*.

Refinement was re-started from the optimized model, and yielded a residual of about 17.39%. Repeated cycling between *GSAS-II* (Toby & Von Dreele, 2013 ▸; Toby, 2026 ▸) and *VASP* did not yield improved models; all had some close contacts, the displacement coefficients refined to large values, and bonds in the alkyl side chains tended to break unless the restraint weights were raised to high values. Poor agreement of the refined and optimized structures was obtained, with r.m.s. differences > 1.0 Å.

A Le Bail fit yielded *R*_wp_ = 0.0718, so we were confident in the unit cell, space group, and profile coefficients. In the Rietveld fits, the largest errors tended to occur at *0kl* peaks with relatively large *l* indices. An optical micrograph indicated the presence of some large crystallites, so the presence of preferred orientation and/or granularity effects had to be considered (Salazar *et al.*, 2026*d* ▸). A better structural model was actually obtained using laboratory data, which (because of the grater divergence) suffered less from granularity effects.

### Dordaviprone di­hydro­chloride hydrate

Dordaviprone hydro­chloride, C_24_H_28_N_4_OCl_2_, (known as ONC201) is used to treat diffuse midline glioma. Powder data for dordaviprone di­hydro­chloride and a nicotinamide co-crystal have been reported by Annereau *et al.* (2024 ▸).

The pattern, measured at 11-BM, was indexed on a primitive monoclinic unit cell with *a* = 7.00566, *b* = 31.19567, *c* = 22.96548 Å, *β* = 97.43°, and *V* = 4976.93 Å^3^ using *JADE Pro* (MDI, 2025 ▸). The volume corresponds to *Z* = 8, so there are two independent cations and four Cl anions in the asymmetric unit. The suggested space group was *P*2_1_/*n*, which was confirmed by successful solution and refinement of the structure. A reduced cell search in the Cambridge Structural Database (version 2026.1.0; Groom *et al.*, 2016 ▸) yielded one hit, but no structures of dordaviprone or its derivatives.

The structure was solved by parallel tempering techniques as implemented in *FOX* (Favre-Nicolin & Černý, 2002 ▸) using two dordaviprone mol­ecules and four Cl atoms as fragments, with [100] preferred orientation. One of the 55 solutions had a cost factor much lower than the others, so was adopted for refinement. [The *FOX* cost factor is a positive number that indicates the quality of the fit of the calculated powder pattern to the experimental data (lower is better).]

Since N3, N5, N58, and N60 atoms (in our numbering) were protonated in the structure of the ethanol solvate of dordaviprone di­hydro­chloride (Wagner *et al.*, 2014 ▸), H atoms were added to these atoms using *Materials Studio* (Dassault Systèmes, 2025 ▸). The solution yielded three Cl anions at reasonable positions, but the fourth was too close to a cation. The ‘missing’ Cl was near N3, so it was placed on the N3–H115 vector, 3.1 Å from N3. The structure contained two voids (*Mercury*/Display/Voids (Macrae *et al.*, 2020 ▸), with probe radius = 1.2 Å): at 0.250, 0.296, 0.200 and 1/2, 0,0. Oxygen atoms (water mol­ecules) O119 and O120 were placed at these voids.

The largest errors were at the 021, 040, and 051 peaks, suggesting granularity. A photomicrograph of the sample revealed the presence of large needles (some of which could probably have been used for a single-crystal structure determination). Since the greater divergence of a laboratory instrument should yield reduced granularity effects, a refinement was also carried out using data collected on a Bruker D2 diffractometer at ICDD Headquarters. A reasonable refinement was obtained (*R*_wp_ = 0.2042, GOF = 1.47, but the texture index calculated from the 4th-order spherical harmonic coefficients was 2.08, consistent with the needle morphology (Salazar *et al.*, 2026*e* ▸).

## Sample prep

The careful reader will have noticed that several of these problem samples had specimen preparation issues, mainly orientation and/or granularity, as well as impurities and a nearly empty capillary. Specimen prep issues never go away, even though some of them can be modeled. It is always worth minimizing them before collecting data.

**Figure 15:**
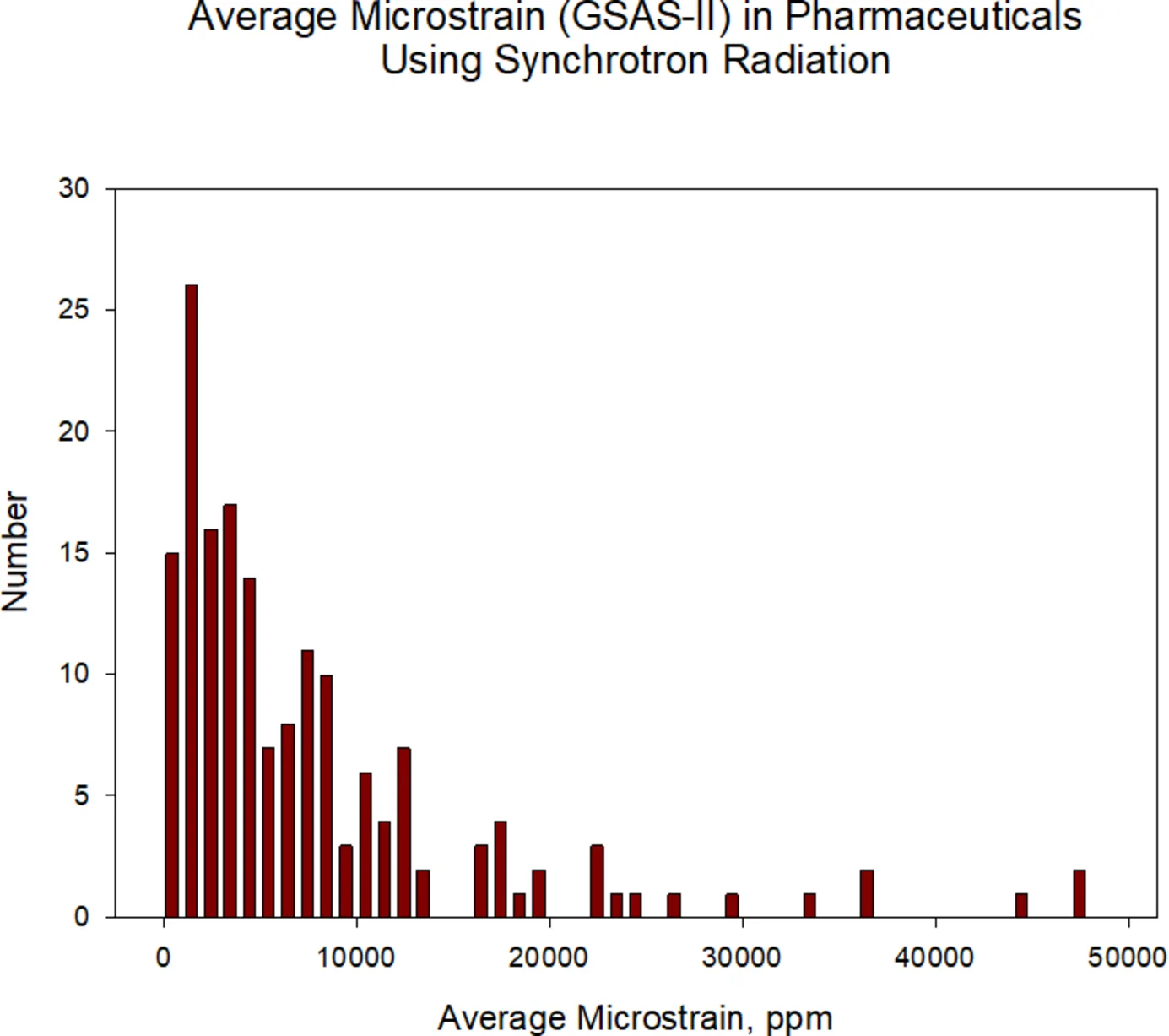
Average microstrain in pharmaceutical structures determined using synchrotron radiation.

But sometimes the issue is with the sample itself. My experience is that the peak profiles of pharmaceuticals are almost always dominated by microstrain broadening. The broadening can vary a great deal among samples (Fig. 15 ▸). Peak broadening increases the degree of overlap, and thus causes a loss of information. The compounds with broader peaks tend to be more difficult to solve and refine. The aim of this project is to characterize materials actually encountered in commerce, but sometimes acquisition or preparation of a better sample is justified.

## Conclusions

When indexing a powder pattern it is often (at least initially) useful to ignore the very weakest peaks (say *I*_rel_ < 1%). If the proposed unit cell accounts for these weak peaks, it lends confidence to the cell. It is often a good strategy to include the possibility of at least one unindexed peak, as there always seems to be a peak which is located less accurately than the others. (Often this apparently single peak is actually a multiplet, and thus yields peak position errors.) Only when these strategies do not yield an acceptable cell should one consider the possibility of more unindexed peaks, and that the sample is really a mixture.

From a Monte Carlo simulated annealing run, it is not always the ‘best’ solution (the lowest residual) that is the correct structure; sometimes the correct structure is farther down the list of suggested solutions.

When assessing the quality of a structure solution or refinement, we consider the statistical residuals (Table 1 ▸) as well as the quality of the graphical fit to the pattern. Most of these problems are large for powder diffraction data, so the residuals might be higher than desired. Even more important is the chemical reasonableness of the model. A mol­ecule should not overlap itself (in a strange conformation) or adjacent mol­ecules. As with daprodustat, sometimes the structure solution can yield a false minimum conformation, and a portion of the mol­ecule may need to be manually rotated to result in more reasonable hydrogen bonds.

The material in the vial is not always the correct compound! Although it is tempting to try to solve the problem completely using only the powder diffraction data, it is OK to ask for help, and use other analytical techniques to confirm the identity of the compound.

## Supplementary Material

A ZIP file containing the final GSAS-II project file for each example. DOI: 10.1107/S2056989026009382/oi2043sup10.zip

The final CIF for defactinib, with the raw data. DOI: 10.1107/S2056989026009382/oi2043sup2.txt

Supporting information file. DOI: 10.1107/S2056989026009382/oi2043sup3.txt

Supporting information file. DOI: 10.1107/S2056989026009382/oi2043sup4.txt

Supporting information file. DOI: 10.1107/S2056989026009382/oi2043sup5.txt

Supporting information file. DOI: 10.1107/S2056989026009382/oi2043sup6.txt

Supporting information file. DOI: 10.1107/S2056989026009382/oi2043sup7.txt

Supporting information file. DOI: 10.1107/S2056989026009382/oi2043sup8.txt

Supporting information file. DOI: 10.1107/S2056989026009382/oi2043sup9.txt

## Figures and Tables

**Figure 2:**
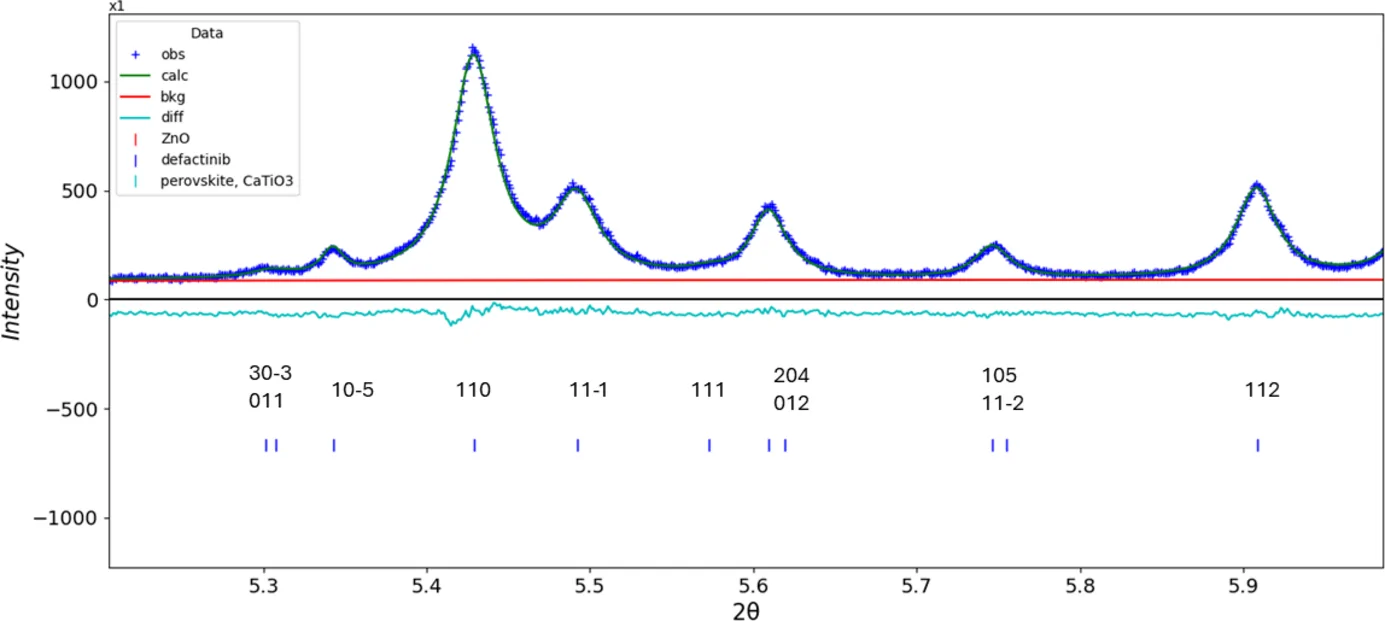
A small portion of the Rietveld plot for defactinib, with the Miller indices of the reflections/peaks.

**Table 1:** Crystallographic summary (Å, °, Å^3^) The texture index is calculated from the refined spherical harmonic coefficients; a value of 1.0 indicates a random sample, and larger values indicate increasing degrees on non-randomness. In the last column, the top numbers are calculated using the *Mercury* CSD-Materials/Search/Crystal Packing Similarity tool, and the bottom numbers are calculated using the *Mercury* Calculate/Mol­ecule Overlay tool.

Compound	space group	cell	*Z*/*Z*′	*R*_wp_	GOF	Restraints constraints	Texture index
Acoltremon	*P*2_1_2_1_2_1_	*a* = 9.320220 (15)	4/1	0.13518	3.59	53	1.034
		*b* = 11.391105 (27)				5	
		*c* = 16.26284 (4)					
		*V* = 1726.587 (5)					
Atrasentan (lab data)	*P*2_1_2_1_2_1_	*a* = 8.0278 (13)	4/1	0.22433	2.82	97	2.482
		*b* = 17.682 (5)				0	
		*c* = 21.363 (4)					
		*V* = 3032.4 (14)					
Bromfenac	*P* 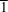	*a* = 4.177042 (8)	4/2	0.07019	1.77	104	1.038
		*b* = 18.31136 (13)				8	
		*c* = 20.31125 (17)					
		α = 98.7657 (9)					
		β = 92.06845 (21)					
		γ = 90.41030 (4)					
		*V* = 1534.281 (4)					
Daprodustat	*P* 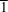	*a* = 6.53821 (9)	4/2	0.09997	1.32	146	1.035
		*b* = 13.76324 (18)				8	
		*c* = 22.2158 (7)					
		α = 88.981 (5)					
		β = 83.8947 (17)					
		γ = 88.8464 (4)					
		*V* = 1987.15 (3)					
Defactinib	*P*2_1_/*n*	*a* = 17.37114 (27)	4/1	0.06628	1.64	93	1.043
		*b* = 5.16713 (6)				7	
		*c* = 25.4639 (4)					
		β = 97.7305 (8)					
		*V* = 2264.84 (6)					
Dordaviprone	*P*2_1_/*n*	*a* = 7.00482 (8)	8/2	0.13208	3.21	166	1.025
		*b* = 31.19722 (18)				7	
		*c* = 22.9596 (5)					
		β = 97.4097 (12)					
		*V* = 4975.49 (4)					
Resmetirom	*P* 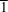	*a* = 11.3094 (23)	4/2	0.07746		158	1.549
		*b* = 15.158 (6)				11	
		*c* = 16.570 (7)					
		α = 67.405 (13)					
		β = 74.425 (7)					
		γ = 69.526 (7)					
		*V* = 2427.2 (4)					
Xanomeline tartrate	*P*2_1_2_1_2_1_	*a* = 7.49025 (19)	4/1	0.07819		67	1.086
		*b* = 7.50712 (10)				5	
		*c* = 37.5187 (9)					
		*V* = 2109.68 (9)					
